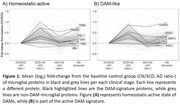# Novel microglial markers that are specifically increased in early clinical AD stages

**DOI:** 10.1002/alz.090758

**Published:** 2025-01-03

**Authors:** Elena Raluca Blujdea, Lisa Vermunt, Alice Chen‐Plotkin, David J Irwin, Walter Boiten, Yolande A.L. Pijnenburg, Wiesje M. van der Flier, Pieter Van Bokhoven, Marta del Campo, Charlotte Teunissen

**Affiliations:** ^1^ Neurochemistry Laboratory, Department of Laboratory Medicine, Vrije Universiteit Amsterdam, Amsterdam UMC, Amsterdam Netherlands; ^2^ Neurochemistry Laboratory, Department of Laboratory medicine, Vrije Universiteit Amsterdam, Amsterdam UMC location VUmc, Amsterdam Netherlands; ^3^ Alzheimer Center Amsterdam, Neurology, Vrije Universiteit Amsterdam, Amsterdam UMC location VUmc, Amsterdam, North Holland Netherlands; ^4^ Perelman School of Medicine, University of Pennsylvania, Philadelphia, PA USA; ^5^ University of Pennsylvania, Philadelphia, PA USA; ^6^ Amsterdam UMC, Amsterdam Netherlands; ^7^ Alzheimer Center Amsterdam, Neurology, Vrije Universiteit Amsterdam, Amsterdam UMC, Amsterdam Netherlands; ^8^ Alzheimer Center Amsterdam, Neurology, Vrije Universiteit Amsterdam, Amsterdam UMC location VUmc, Amsterdam Netherlands; ^9^ Industry Alliance Office, Amsterdam Neuroscience, Amsterdam UMC, Amsterdam Netherlands; ^10^ Barcelonaβeta Brain Research Center (BBRC), Pasqual Maragall Foundation, Barcelona Spain; ^11^ Neurochemistry Laboratory, Department of Clinical Chemistry, Vrije Universiteit Amsterdam, Amsterdam UMC location VUmc, Amsterdam, North Holland Netherlands

## Abstract

**Background:**

Transcriptomic and pathological studies indicate that microglia play a key role in the progression of Alzheimer’s disease (AD). Throughout the stages of the AD continuum, there may be varying microglia phenotypes, such as the disease‐associated microglia (DAM). Microglia proteins have been detected in cerebrospinal‐fluid (CSF), providing a quantifiable avenue for potential stage‐detection. This study aims to find CSF microglial biomarkers to stage early‐ versus late‐AD.

**Method:**

In total, 637 proteins were measured with proximity extension assay in 564 samples from the Amsterdam Dementia Cohort and from University of Pennsylvania. We defined four clinical groups spanning the AD continuum, as follows: 1) cognitively normal or subjective cognitive decline with negative AD‐ratio of CSF t‐tau/amyloid‐beta_42_ as controls, and positive AD‐ratio 2) subjective cognitive decline, 3) mild cognitive impairment, and 4) AD‐dementia. We selected 176 microglia‐enriched proteins based on human‐derived single‐cell RNA‐sequencing data and compared the groups by ANCOVA and Tukey’s HSD. Functional characterisation was done by associating proteins of interest to a list of microglial markers per state, such as DAM‐like proteins, that were identified through single‐cell Hierarchical Poisson Factorization.

**Results:**

Overall, proteins showed distinct wave‐like patterns of dysregulation, presented in figure 1. 21 of the 176 microglial proteins were specifically upregulated in the early stages, while 20 were specific to the dementia stage. The proteins increased specifically in early stages consisted of receptors, possibly reflecting increased cellular recruitment, with an increase in DAM‐like proteins, particularly ones associated to MHC class II binding, such as CD4 and CD74, which are related to adaptive innate immune response.

**Conclusion:**

In conclusion, we identify varying microglial protein signatures spanning the AD stages, which may provide a basis for early‐staging biomarkers. Independent cohort and cross‐method validations are ongoing, and are necessary for the understanding of the hypothesized dynamicity of AD inflammation driven by microglia.